# IL‐1β Is an Androgen‐Responsive Target in Macrophages for Immunotherapy of Prostate Cancer

**DOI:** 10.1002/advs.202206889

**Published:** 2023-04-24

**Authors:** Deng Wang, Chaping Cheng, Xinyu Chen, Jinming Wang, Kaiyuan Liu, Na Jing, Penghui Xu, Xialian Xi, Yujiao Sun, Zhongzhong Ji, Huifang Zhao, Yuman He, Kai Zhang, Xinxing Du, Baijun Dong, Yuxiang Fang, Pengcheng Zhang, Xueming Qian, Wei Xue, Wei‐Qiang Gao, Helen He Zhu

**Affiliations:** ^1^ State Key Laboratory of Oncogenes and Related Genes Renji‐Med‐X Stem Cell Research Center Shanghai Cancer Institute & Department of Urology Ren Ji Hospital School of Medicine and School of Biomedical Engineering Shanghai Jiao Tong University Shanghai 200127 P. R. China; ^2^ School of Biomedical Engineering and Med‐X Research Institute Shanghai Jiao Tong University Shanghai 200030 P. R. China; ^3^ Department of Urology Ren Ji Hospital Shanghai Jiao Tong University School of Medicine Shanghai 200127 P. R. China; ^4^ School of Biomedical Engineering ShanghaiTech University Shanghai 201210 P. R. China; ^5^ Mabspace Biosciences (Suzhou) Co. Limited Suzhou 215123 P. R. China

**Keywords:** IL‐1β, androgen deprivation therapy, androgen receptor, immune therapy, prostate cancer, tumor‐associated macrophage

## Abstract

Great attention is paid to the role of androgen receptor (AR) as a central transcriptional factor in driving the growth of prostate cancer (PCa) epithelial cells. However, the understanding of the role of androgen in PCa‐infiltrated immune cells and the impact of androgen deprivation therapy (ADT), the first‐line treatment for advanced PCa, on the PCa immune microenvironment remains limited. On the other hand, immune checkpoint blockade has revolutionized the treatment of certain cancer types, but fails to achieve any benefit in advanced PCa, due to an immune suppressive environment. In this study, it is reported that AR signaling pathway is evidently activated in tumor‐associated macrophages (TAMs) of PCa both in mice and humans. AR acts as a transcriptional repressor for *IL1B* in TAMs. ADT releases the restraint of AR on *IL1B* and therefore leads to an excessive expression and secretion of IL‐1β in TAMs. IL‐1β induces myeloid‐derived suppressor cells (MDSCs) accumulation that inhibits the activation of cytotoxic T cells, leading to the immune suppressive microenvironment. Critically, anti‐IL‐1β antibody coupled with ADT and the immune checkpoint inhibitor anti‐PD‐1 antibody exerts a stronger anticancer effect on PCa following castration. Together, IL‐1β is an important androgen‐responsive immunotherapeutic target for advanced PCa.

## Graphical abstract

Androgen receptor signaling is active in tumor‐associated macrophages (TAMs) and inhibits the transcription of IL‐1β in prostate cancer. Androgen deprivation therapy (ADT) promotes the production of IL‐1β by TAMs, leading to enhanced myeloid‐derived suppressor cells recruitment and immune therapy resistance. Targeting IL‐1β and blocking immune checkpoints in combination with ADT could be a promising therapeutic approach for advanced prostate cancer.

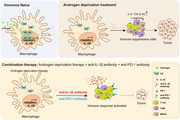

## 1 Introduction

Prostate cancer (PCa) is one of the most frequent types of cancer and a leading cause of cancer‐related death among males worldwide.^[^ ^1^ ^]^ Androgen deprivation therapy (ADT), categorized into luteinizing hormone‐releasing hormone (LHRH) agonists, LHRH antagonists and androgen receptor (AR) antagonist, has generated impressive effects in treating advanced PCa.^[^ ^2^ ^]^ However, even with the application of potent second‐generation antiandrogen agents including enzalutamide and abiraterone, the most majority of PCa patients suffer from disease relapse inevitably.^[^ ^2^, ^3^ ^]^New treatment options or novel combination therapies in addition to ADT are urgently needed for advanced PCa.

AR is a master transcription factor and driver in PCa. Androgens such as testosterone and dihydrotestosterone (DHT) bind to AR in the cytoplasm and trigger the dissociation of heat shock proteins from AR.^[^ ^4^ ^]^ The activated AR translocates into the nucleus and binds to androgen response elements in target genes for subsequent transcription regulation.^[^ ^4^, ^5^ ^]^ In addition to epithelial cells, several environmental cell types in PCa and other solid tumors have been demonstrated to be responsive to androgen.^[^ ^6^ ^]^ It is reported that AR in cancer‐associated fibroblasts (CAFs) has a more dominant tumor‐promoting function than that in epithelial cells at the early stage of PCa development.^[^ ^6a^ ^]^ In addition, reduction of AR in CAFs is demonstrated to cooperate with CSL/RBP‐J*κ* to facilitate transcriptional activation of CAFs effector genes, which in turn promotes the progression of squamous cell carcinoma and melanoma.^[^ ^6b^ ^]^ Therefore, ADT could incur systemic consequences besides the inhibitory effect on PCa epithelial cells, which is likely to contribute to treatment resistance and tumor recurrence. However, the impacts of ADT on environmental cells, especially the tumor‐associated immune cells, remains to be largely unexplored.

The immune checkpoint blockade (ICB) therapy, such as anti‐PD‐1 and anti‐PD‐L1 antibodies, which boosts the cellular immune response by antagonizing bindings between suppressive receptors on T cells and their respective ligands, has achieved a groundbreaking success in treating melanoma, lymphoma and some solid tumors.^[^ ^7^ ^]^ However, prostate cancer, a typical cold tumor that is scarcely infiltrated with cytotoxic T cells, is found to respond poorly or is completely refractory to ICB.^[^ ^8^ ^]^ Recent clinical trials have shown that even combinatory therapy of ADT and ICB generates minimal effect on human PCa.^[^ ^9^ ^]^ Further understanding of key immune suppressive components and identification of new immune therapy targets will be a prerequisite to increase the responding rate in PCa to ICB.

Myeloid cells, especially tumor‐associated macrophages (TAMs), are one of the most abundant immune cell types in PCa.^[^ ^10^ ^]^ Previous studies have shown that TAMs inhibit the proliferation of CD8^+^ T cells in breast cancer,^[^ ^11^ ^]^ induce PD‐L1 expression to inhibit antitumor activity of T cells in hepatocellular carcinoma,^[^ ^12^ ^]^ and recruit regulatory T cells to tumor microenvironment in colorectal cancer and melanoma,^[^ ^13^ ^]^ thereby exert immunosuppressive effects that limit the efficacy of ICB. Interleukin 1 beta (IL‐1β), a member of the interleukin 1 cytokine family, is produced by myeloid cells such as macrophages as a preprotein that is proteolytically processed by caspase 1 into its active form.^[^ ^14^ ^]^ IL‐1β is an important mediator of the inflammatory response, which promotes the recruitment of monocytes at sites of inflammation by inducing the expression of adhesion molecules such as ICAM‐1 and VCAM‐1 on endothelial cells.^[^ ^14^, ^15^ ^]^ Besides its function in inflammation, IL‐1β is proposed to contribute to an immune suppressive microenvironment in gastric and breast cancer.^[^ ^16^ ^]^ However, whether and how ADT affects TAMs and their immunosuppressive functions in PCa remains to be elucidated.

Here, we report that TAMs in PCa are androgen‐responsive. IL‐1β, which is suppressed by AR in TAMs, is unleashed by ADT and causes an aggravation of the immunosuppressive microenvironment in PCa. Anti‐IL‐1β antibody exerts a tumor‐inhibitory impact and displays a synergistic effect with ADT and ICB on advanced PCa.

## 2 Results

### 2.1 Androgen Deprivation Therapy Rewires the Immune Microenvironment in Prostate Cancer

**Figure 1:**
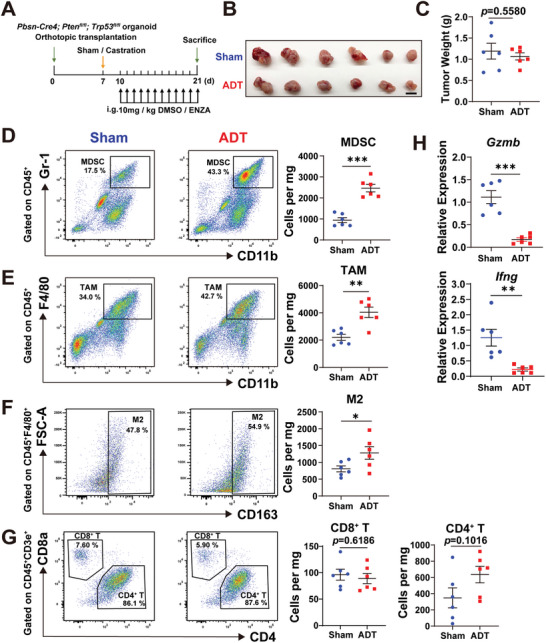
Androgen deprivation therapy reshapes the tumor immune microenvironment in prostate cancer. A) Schematic illustration of experimental design. Prostate cancer organoids derived from the *Pbsn‐Cre4*; *Pten* ^fl/fl^; *Trp53* ^fl/fl^ mouse model were orthotopically implanted to C57BL/6J mice. Recipient mice were sham operated (*n* = 6) or castrated (*n* = 6) at day 7, and administrated with enzalutamide (i.g., 10 mg kg^−1^) or vehicle daily from day 10. On day 21, animals were sacrificed for tissue collection and analysis. B) Prostate tumor image (scale bar = 1 cm) and C) weight of control and ADT treated mice. D—G) Gating strategies for analysis of the tumor immune microenvironment by flow cytometry (left panel). Number of D) MDSCs, E) TAMs, F) M2 TAMs, G) CD8^+^ T cells and CD4^+^T cells per mg of tumor weight (cells per mg) (right panel). H) qRT–PCR analyses of *Gzmb* and *Ifng* mRNA levels in CD8^+^ T cells. Gene expression was normalized to the expression of *Actb*. (Two‐tailed Student's *t* test was used for the statistical analysis. *, *p* < 0.05; **, *p* < 0.01; ***, *p* < 0.001. Data are presented as means ± SEM).

To assess effects of androgen deprivation therapy (ADT) on the immune microenvironment of PCa, we implanted PCa organoids derived from the *Pbsn‐Cre4*; *Pten* ^fl/fl^; *Trp53* ^fl/fl^ (*Pten* ^Δ/Δ^; *Trp53* ^Δ/Δ^) genetically engineered mouse model (GEMM) to C57BL/6J mice orthotopically and subjected the recipient mice to castration and enzalutamide treatment (**Figure** **1**A). We found that ADT did not ameliorate the in vivo growth of *Pten* ^Δ/Δ^; *Trp53* ^Δ/Δ^ PCa compared to the sham operated group, consistent with the previous study that *Pbsn‐Cre4*; *Pten* ^fl/fl^; *Trp53* ^fl/fl^ mice develop aggressive castration resistance PCa^[^ ^17^ ^]^ (Figure 1B,C). Tumors were collected for flow cytometry assay to characterize the impact of ADT on different type of immune cells (Figure 1D–G and Figure S1, Supporting Information). As shown in Figure 1G, we did not observe a significant difference in the numbers of CD8^+^ T cells and CD4^+^ T cells between sham and ADT‐treated mice. However, the RNA levels of *Gzmb* and *Ifng* in sorted tumor‐infiltrated CD8^+^ T cells from the ADT‐treated group were markedly lower than those in the sham‐operated group (Figure 1H), suggesting that ADT suppresses the cytotoxic function of CD8^+^ T cells. Intriguingly, we found significant alterations in myeloid cell populations. The number of myeloid‐derived suppressor cells (MDSCs) in the ADT group was notably higher than that in the sham‐operated group (Figure 1D). In addition, tumor‐associated macrophages (TAMs) and tumor‐promoting M2‐type TAMs in the ADT group were significantly increased than those in the control group (Figure 1E,F). These results suggest that androgen deprivation therapy remodels the immune microenvironment of PCa and exacerbates immunosuppression.

### 2.2 IL‐1β is Markedly Upregulated in ADT‐Treated Murine Prostate Cancers

**Figure 2:**
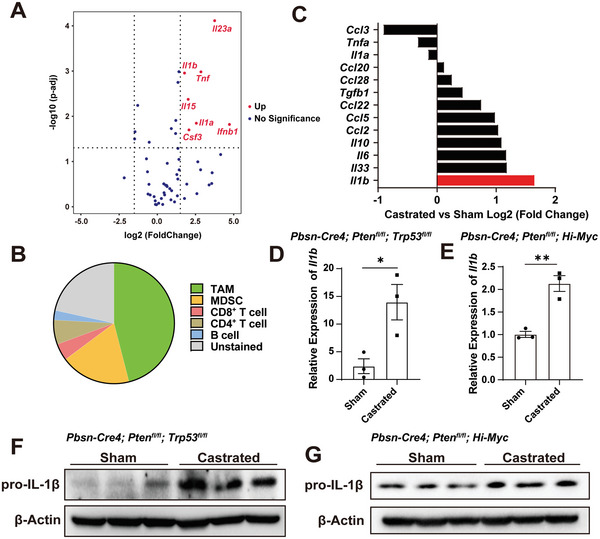
IL‐1β is a top upregulated cytokine in tumor‐associated macrophages of prostate cancer after androgen deprivation therapy. A) Differential expression of immunosuppressive genes from RNA‐seq in castrated *Pbsn‐Cre4*; *Pten* ^fl/fl^; *Trp53* ^fl/fl^ GEMMs prostate tumors compared to sham operated isotype control mice. Data are shown as pool of *n* = 3. B) Pie chart showing the proportion of TAM, MDSC, CD8^+^ T cells, CD4^+^ T cells, B cells in CD45^+^ cells from *Pbsn‐Cre4*; *Pten* ^fl/fl^; *Trp53* ^fl/fl^ organoid‐derived tumors. Data are shown as pool of *n* = 3. C) Relative mRNA expression of cytokines in TAMs sorted from *Pbsn‐Cre4*; *Pten* ^fl/fl^; *Trp53* ^fl/fl^ GEMMs prostate tumors. Data are shown as pool of *n* = 4. D,E) qRT–PCR analyses of *Il1b* in prostate cancer tissues from sham‐operated or castrated D) *Pbsn‐Cre4*; *Pten* ^fl/fl^; *Trp53* ^fl/fl^ mice (*n* = 3) or E) *Pbsn‐Cre4*; *Pten* ^fl/fl^; *Hi‐Myc* mice (*n* = 3). Gene expression was normalized to *Actb*. F,G) The protein levels of IL‐1β in sham‐operated and castrated F) *Pbsn‐Cre4*; *Pten* ^fl/fl^; *Trp53* ^fl/fl^ mice or G) *Pbsn‐Cre4*; *Pten* ^fl/fl^; *Hi‐Myc* mice were determined by immunoblotting (*n* = 3). (Two‐tailed Student's *t*‐test was used for the statistical analysis. *, *p* < 0.05; **, *p* < 0.01. Data are presented as means ± SEM).

Cytokines play essential roles in shaping the cancer immune microenvironment. To decipher the impacts of ADT on expression levels of cytokines in PCa, we performed RNA sequencing of PCa tissues from sham versus castrated *Pbsn‐Cre4*; *Pten* ^fl/fl^; *Trp53* ^fl/fl^ mice. As shown in **Figure** **2**A, ADT significantly reprogrammed the cytokine expression pattern as a robust number of cytokine coding genes were enriched in the differentially expressed genes (DEGs) in PCa upon ADT (Figure S2, Supporting Information). Of note, we carefully examined the immune cell components within *Pten* ^Δ/Δ^; *Trp53* ^Δ/Δ^ PCa and found that TAMs represented the most abundant subpopulation in CD45^+^ immune cells (Figure 2B). Therefore, we sorted the TAMs from PCa tissues from control and castrated *Pbsn‐Cre4*; *Pten* ^fl/fl^; *Trp53* ^fl/fl^ mice to analyze the expression alterations of cytokines identified from the DEGs of RNA‐seq. As shown in Figure 2C, we found that IL‐1β was ranked as the most upregulated cytokine in TAMs of PCa following ADT. Using immunoblotting and qRT‐PCR, we further verified that IL‐1β was markedly increased in castrated *Pten* ^Δ/Δ^; *Trp53* ^Δ/Δ^ prostate cancers (Figure 2D,F). Also, we found that the expression of IL‐1β was significantly upregulated after castration in *Pbsn‐Cre4*; *Pten* ^fl/fl^; *Hi‐Myc* prostate tumors (Figure 2E,G). These data demonstrate that IL‐1β is upregulated after ADT in murine PCa.

### 2.3 AR is Expressed in Human PCa‐Associated Macrophages and Negatively Correlates with IL‐1β

**Figure 3:**
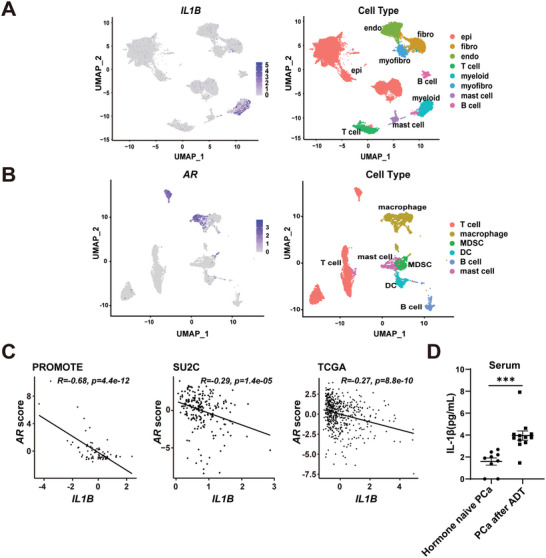
AR is expressed in tumor‐associated macrophages of human prostate cancer and negatively correlates with IL‐1β. A,B) Visualization of *IL1B* gene expression in different cell types (A) and *AR* gene expression in CD45^+^ cells (B) on a UMAP plot of scRNA‐seq profiles of human prostate cancers. Data were obtained from Baijun Dong et al.^[^ ^18^ ^]^ C) Correlation analysis of *IL1B* mRNA level and AR score in the PROMOTE 2017 PCa dataset,^[^ ^19^ ^]^ SU2C 2019 PCa dataset^[^ ^20^ ^]^ and TCGA 2018 PCa dataset.^[^ ^21^ ^]^ Pearson Correlation Coefficient was used for the correlation analysis. D) The concentrations of IL‐1β in the serum of patients with hormone naïve PCa (*n* = 9) or PCa after ADT (*n* = 12) was measured by enzyme‐linked immunosorbent assay. (Two‐tailed Student's *t*‐test was used for the statistical analysis. ***, *p* < 0.001. Data are presented as means ± SEM).

Next, we set force to determine the major secreting cell type of IL‐1β in human PCa. We processed the published human PCa single‐cell RNA‐seq data for dimensionality reduction clustering.^[^ ^18^ ^]^ *IL1B* was found to be mainly expressed in myeloid cells (**Figure** **3**A). We then further examined the subpopulation within the CD45^+^ immune cells. Interestingly, expression of *AR* was relatively enriched in TAMs compared to other immune cells (Figure 3B). As shown in Figure 2D–G, we found that the expression level of IL‐1β was significantly increased after ADT treatment in murine PCa. Therefore, we investigated several human PCa RNA‐seq datasets (including the PROMOTE 2017 PCa dataset by Wang L et al.,^[^ ^19^ ^]^ the SU2C 2019 PCa dataset by Abida W et al.,^[^ ^20^ ^]^ and the TCGA 2018 PCa dataset by Hoadley KA et al.^[^ ^21^ ^]^), and found a negative correlation between the *IL1B* transcription level and the AR score (Figure 3C). We then performed an enzyme‐linked immunosorbent assay (ELISA) to measure serum IL‐1β in human PCa patients. As shown in Figure 3D, serum IL‐1β concentrations were significantly higher in PCa samples following ADT than in hormone naive PCa patients. Collectively, these data suggest that IL‐1β is inversely correlated with the AR signaling activity in human PCa.

### 2.4 AR Acts as a Transcriptional Suppressor of *Il1b* in PCa‐Associated Macrophages

**Figure 4:**
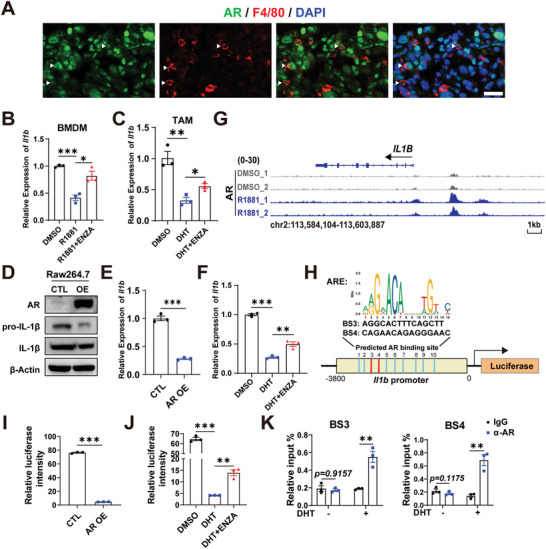
AR acts as a transcriptional suppressor for *Il1b* in tumor‐associated macrophages of prostate cancer. A) Immunofluorescent staining images of AR and F4/80 in *Pbsn‐Cre4*; *Pten* ^fl/fl^; *Trp53* ^fl/fl^ GEMMs prostate tumors. Representative images are presented. Scale bars = 20 µm. B,C) qRT–PCR analyses of *Il1b* in bone marrow derived macrophages (BMDMs) (B) and TAMs (C) treated with DMSO, 10 nm DHT, 10 nm DHT plus 10 µm enzalutamide, respectively. TAMs were FACS‐sorted from *Pten* ^Δ/Δ^; *Trp53* ^Δ/Δ^ organoids‐derived tumor. D,E) Immunoblotting (D) and qRT–PCR (E) results showing IL‐1β protein (D) and mRNA (E) levels in control and AR overexpressed (OE) Raw264.7 macrophage cell line. Gene expression was normalized to the expression of *Actb*. F) qRT–PCR analyses of *Il1b* in Raw264.7 treated with DMSO, 10 nm DHT, 10 nm DHT plus 10 µm enzalutamide, respectively. G) Genome views of AR enrichment at the *IL1B* gene in THP‐1 cells from analysis of published Chip‐seq data (Bianca Cioni et al.)^[^ ^22^ ^]^ (Scale bar = 1 kb). AR peaks in DMSO and 10 nm R1881 conditions are depicted in grey and blue, respectively. H) Predicted AR binding sites on the promoter of *Il1b* by the JASPAR database. I,J) Relative luciferase intensity of *Il1b* promoter‐driven firefly luciferase in control and AR OE Raw264.7 cell lines (I), and in Raw264.7 cells treated with DMSO, 10 nm DHT, 10 nm DHT and 10 µm enzalutamide, respectively (J). The firefly luciferase signal was normalized to the co‐transfected renilla signal. K. ChIP‐q‐PCR analysis shows enrichment levels of AR to the *Il1b* promoter in Raw264.7 cells without or with the treatment of 10 nm DHT. (Two‐tailed Student's *t*‐test was used for the statistical analysis: ns, not significant; *, *p* < 0.05; **, *p* < 0.01; ***, *p* < 0.001. Data are presented as means ± SEM).

In light of the negative relationship between AR and IL‐1β, both of which were found to be expressed in TAMs of PCa, we therefore asked whether PCa‐associated macrophages are androgen responsive. First, we confirmed that AR was presented in macrophages from *Pbsn‐Cre4*; *Pten* ^fl/fl^; *Trp53* ^fl/fl^ prostate tumors (**Figure** **4**A). We then treated bone marrow‐derived macrophages (BMDMs) with AR agonist R1881 and AR antagonist enzalutamide to evaluate the effect of AR signaling activity on *Il1b* expression. Notably, the transcription of *Il1b* in bone marrow‐derived macrophages decreased significantly upon AR activation by R1881, and rebounded after enzalutamide treatment (Figure 4B). In addition, similar effects were obtained from FACs‐sorted TAMs in *Pbsn‐Cre*4; *Pten* ^fl/fl^; *Trp53* ^fl/fl^ prostate tumors (Figure 4C). To further explore the regulatory relationship of AR on *Il1b*, we used an AR‐expressing lentivirus to achieve AR upregulation in a macrophage cell line Raw264.7. As shown in Figure 4D,E, AR overexpression repressed the RNA and protein levels of *Il1b* in macrophages. Consistent with the results in BMDMs and TAMs, transcription of *Il1b* was significantly decreased in Raw264.7 following the AR activation by DHT and recovered after the enzalutamide treatment (Figure 4F). In addition, AR knockout in Raw264.7 and macrophage‐like THP‐1 cells led to an increase of the expression of IL‐1β (Figures S3A–D, Supporting Information). The analysis of published ChIP‐seq data^[^ ^22^ ^]^ revealed that treatment with AR agonist R1881 enhanced the binding of AR to *IL1B* promoter in a human macrophage‐like cell line THP‐1 (Figure 4G). Therefore, we asked whether AR, the most important transcription factor in PCa, directly regulates *Il1b* at the transcriptional level. Luciferase reporter assay showed that the *Il1b* promoter‐driven luciferase intensity was significantly reduced upon AR overexpression (Figure 4I). Furthermore, DHT treatment greatly inhibited the murine *Il1b* promoter‐driven luciferase activity, which effect could be compromised by the addition of enzalutamide (Figure 4J). Consistently, similar results were obtained from the human *IL1B* gene promoter‐driven luciferase assay (Figure S3E–G, Supporting Information). We then used the JASPAR database (https://jaspar.genereg.net/) to predict putative AR binding sites of *Il1b* promoter (Figure 4H). We assessed the top ten predicted binding sites (BS) by ChIP‐q‐PCR and found a significant enrichment of AR on BS3 and BS4 of the *Il1b* promoter (Figure 4K). Together, the above results indicate that AR directly inhibits the transcription of *Il1b* in PCa‐associated macrophages, and that the increase of *Il1b* in ADT‐treated PCa is attributable to the release of suppression on *Il1b* by AR.

### 2.5 IL‐1β Promotes Recruitment of MDSCs to Exacerbate Immunosuppression in Prostate Cancer

**Figure 5:**
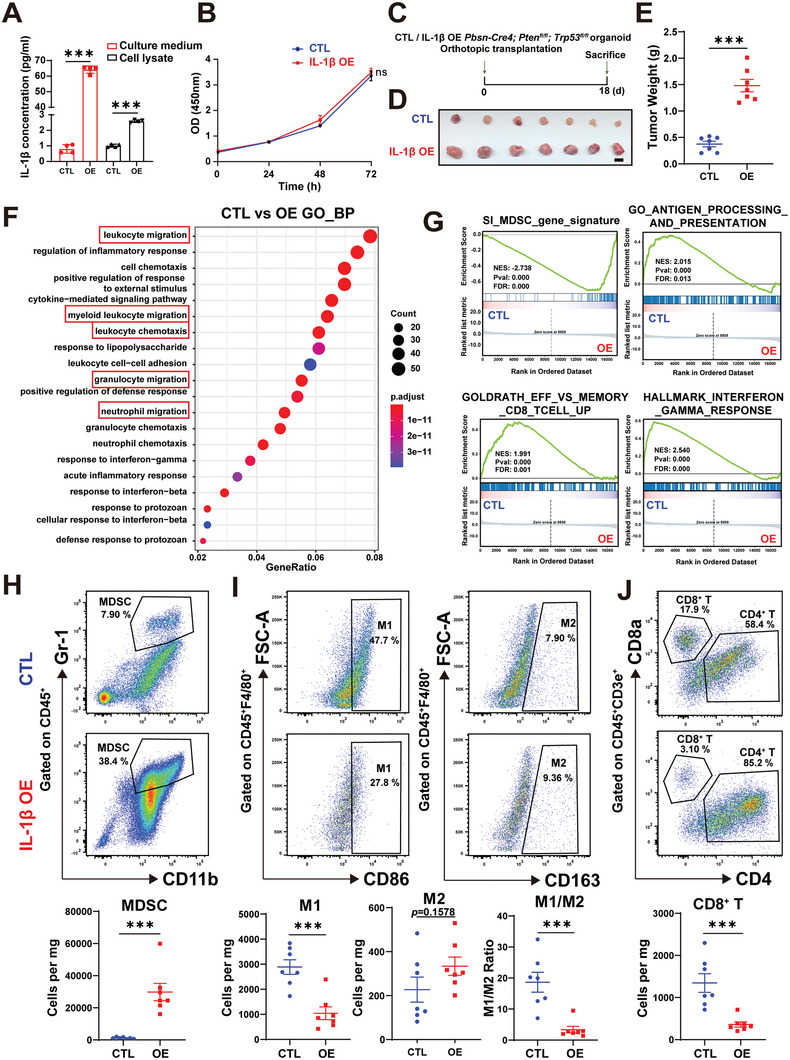
IL‐1β promotes aggregation of myeloid‐derived suppressor cells to exacerbate immunosuppression in prostate cancer. A) IL‐1β concentrations in culture supernatants or cell lysates of control and secreted IL‐1β‐overexpressed *Pten* ^Δ/Δ^; *Trp53* ^Δ/Δ^ PCa cells were measured by enzyme‐linked immunosorbent assay (ELISA). B) CCK‐8 assays showing the growth of control and IL‐1β‐overexpressed *Pten* ^Δ/Δ^; *Trp53* ^Δ/Δ^ PCa cells. C) Schematic illustration of experimental design. Control or secreted IL‐1β‐overexpressed *Pten* ^Δ/Δ^; *Trp53* ^Δ/Δ^ PCa cells were orthotopically implanted to C57BL/6J mice. On day 18, animals were sacrifice for tissue collection and analysis. D,E) Prostate tumor image (D) (Scale bar = 1 cm) and weight (E) of control and IL‐1β‐overexpressed tumors. F) Gene sets enriched in IL‐1β overexpressed tumors compared to control tumors in GO‐biological processing analysis. G) GSEA analysis of RNA‐seq of IL‐1β overexpressed tumors compared to control tumors in MDSC gene signature,^[^ ^23^ ^]^ antigen processing and presentation, effective versus memory CD8^+^ T cell and interferon gamma response. H—J) Gating strategies for analysis of the tumor infiltrated immune cells by flow cytometry (top panel). Number of MDSCs, M1‐type TAMs, M2‐type TAMs, and CD8^+^ T cells normalized to mg of tumor weight (cells per mg) and M1/M2 TAMs ratio in control and IL‐1β‐overexpressed prostate tumors (bottom panel). (Two‐tailed Student's *t*‐test was used for the statistical analysis: ns, not significant; *, *p* < 0.05; **, *p* < 0.01; ***, *p* < 0.001. Data are presented as means ± SEM).

To explore how IL‐1β affects tumor growth, we used a secreted IL‐1β‐expressing lentivirus in *Pten* ^Δ/Δ^; *Trp53* ^Δ/Δ^ PCa cells to achieve intra‐tumoral upregulation of IL‐1β (**Figure** **5**A). As shown in Figure 5B, overexpression of IL‐1β showed minimal effects on tumor cell growth in vitro. In contrast, the overexpression of IL‐1β substantially exacerbated tumor growth in vivo (Figure 5C–E), suggesting that the regulation of tumor growth by IL‐1β is related to the tumor microenvironment. Then, we performed RNA‐seq between the control group and IL‐1β‐overexpressed tumors. Gene Onotology (GO) analysis and Kyoto Encyclopedia of Genes and Genomes (KEGG) analysis showed that overexpression of IL‐1β notably increased myeloid leukocyte migration (Figure 5F), enhanced cytokine responses (Figure S4A, Supporting Information), and highly inhibited antigen presentation (Figure 5G and Figure S4B, Supporting Information). To further explore the underlying mechanism, we performed GSEA analysis and found that overexpression of IL‐1β significantly increased the expression of the MDSC signature,^[^ ^23^ ^]^ while the function of CD8^+^ effector T cells was inhibited (Figure 5G). We then analyzed tumor‐infiltrating immune cells by flow cytometry experiments (Figure 5H–J). Consistent with the analysis of RNA‐seq data, flow cytometry showed that MDSCs increased massively after IL‐1β overexpression (Figure 5H). We found that the ratio of M2‐type TAMs to M1‐type TAMs was increased (Figure 5I). In addition, the infiltration of CD8^+^ T cells was also significantly reduced (Figure 5J). qRT‐PCR results showed that the expression of classical MDSC immunosuppressive factors *Il6*, *Arg1*, *Nos2* was notably increased after IL‐1β overexpression (Figure S4C, Supporting Information), whereas the expression of *Gzmb* and *Ifng* in cytotoxic T cells was downregulated (Figure S4D, Supporting Information). Furthermore, we extracted bone marrow cells from C57BL/6J mice and induced them into bone marrow‐derived MDSCs (BM‐MDSCs) using GM‐CSF and IL6. The immunosuppression‐related molecules, such as *Cd274*, *Il6*, *Arg1*, *Nos2* and *Tgfb1* were significantly upregulated after the recombinant IL‐1β protein treatment (Figure S4E, Supporting Information). Collectively, these data showed that IL‐1β promotes immunosuppression in prostate cancer by inducing the accumulation of MDSCs.

### 2.6 Anti‐IL‐1β Antibody Potentiates the Anti‐Tumor Effect of ADT Plus Anti‐PD‐1 Antibody in Prostate Cancer

**Figure 6:**
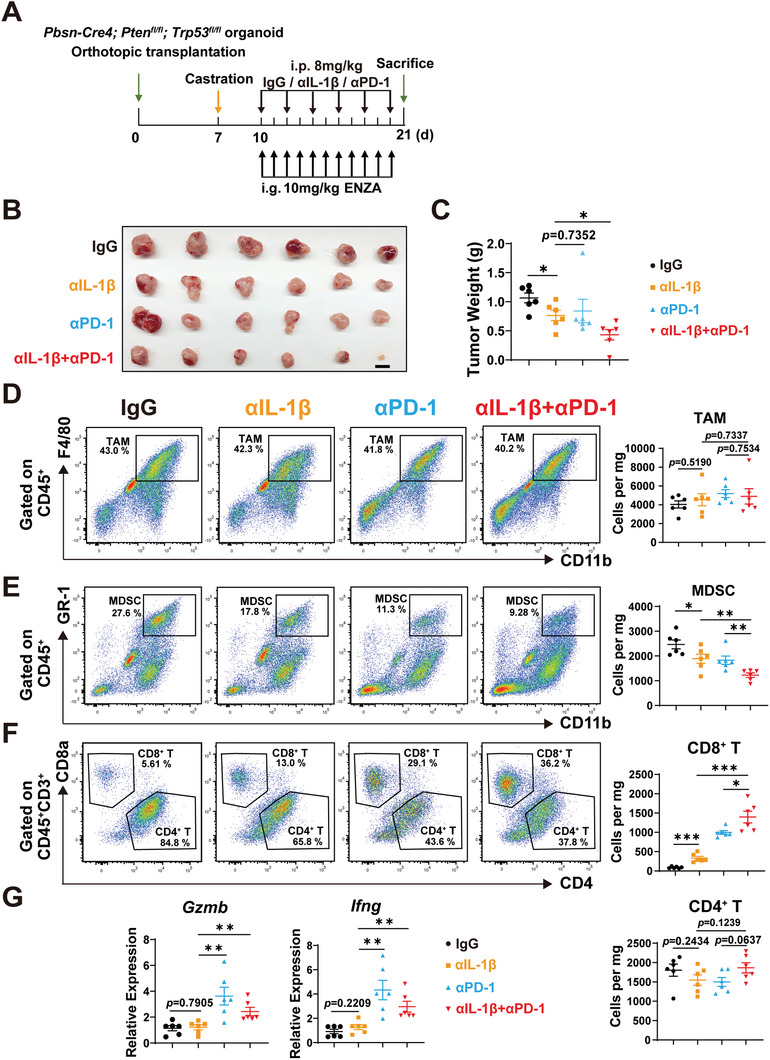
Androgen deprivation therapy combined with anti‐IL‐1β and anti‐PD‐1 immunotherapy significantly inhibits prostate cancer progression. A) Schematic illustration of the treatment strategy on *Pbsn‐Cre4*; *Pten* ^fl/fl^; *Trp53* ^fl/fl^ organoid‐derived tumors. C57BL/6J mice were castrated on day 7 after implantation of tumor organoids, then treated with enzalutamide (i.g., 10 mg kg^−1^) daily from day 10. Mice were arbitrarily divided into four groups and treated with control IgG, anti‐IL‐1β antibody (i.p. 8 mg kg^−1^, every other day), anti‐PD‐1 antibody (i.p. 8 mg kg^−1^, every other day), or anti‐IL‐1β antibody in combination with anti‐PD‐1 antibody. Animals were sacrificed on day 21 for analysis and data collection. B,C) Prostate tumor image (B) (Scale bar = 1 cm) and weight (C) of IgG, anti‐IL‐1β antibody, anti‐PD‐1 antibody, and combinatory antibodies‐treated mice. D–F) Gating strategies for analysis of the tumor infiltrated immune cells by flow cytometry (left panel). Number of TAMs, MDSCs, CD8^+^ T, and CD4^+^ T cells normalized to mg of tumor weight (cells per mg) in prostate tumors of IgG, anti‐IL‐1β antibody, anti‐PD‐1 antibody, and combinatory antibodies‐treated mice (right panel). G) qRT–PCR analyses of *Gzmb* and *Ifng* genes in sorted CD8^+^ T cells from prostate tumors of IgG, anti‐IL‐1β antibody, anti‐PD‐1 antibody, and combinatory antibodies‐treated mice. Gene expression was normalized to the expression of *Actb*. (Two‐tailed Student's *t*‐test was used for the statistical analysis. *, *p* < 0.05; **, *p* < 0.01; ***, *p* < 0.001. Data are presented as means ± SEM).

To determine the effect of IL‐1β upregulation on PCa immune microenvironment and tumor growth, we administrated an IL‐1β blocking antibody on ADT‐treated *Pten* ^Δ/Δ^; *Trp53* ^Δ/Δ^ PCa‐bearing mice (**Figure** **6**A). As shown in Figure 6B,C, treatment with anti‐IL‐1β antibody inhibited the in vivo growth of *Pten* ^Δ/Δ^; *Trp53* ^Δ/Δ^ PCa under the androgen‐deprived condition. We then analyzed tumor‐infiltrating immune cells by flow cytometry (Figure 6D–F). Treatment with anti‐IL‐1β antibody did not affect the number of TAMs (Figure 6D), but reduced the quantity of MDSCs (Figure 6E) and increased the number of tumor‐infiltrating CD8^+^ T cells (Figure 6F). However, function of cytotoxic T cells, characterized by expressional level of *Gzmb* and *Ifng* were not elevated by the administration of anti‐IL‐1β antibody (Figure 6G), which may be caused by a high expression level of immune check protein PD‐1 and PD‐L1 (Figure S5A,B, Supporting Information). Additionally, we applied a newly developed bioinformatic tool TIDE^[^ ^24^ ^]^ to assess the association between IL‐1β levels in PCa and the ICB treatment response. The TIDE value, a parameter predicting the response to ICB, of the IL‐1β overexpression group was much higher than that of the control group. In addition, the TIDE analysis also indicated increases in T cell exclusion potential and MDSC signature (Figure S6, Supporting Information). Therefore, we asked whether an additional usage of anti‐PD‐1 antibody, which can block the inhibitory signal by immune checkpoint molecules in cytotoxic T cells, could achieve a better inhibitory effect on prostate cancer (Figure 6A). As shown in Figure 6B,C, the combination of anti‐IL‐1β antibody and anti‐PD‐1 antibody could notably alleviate the tumor growth of *Pten* ^Δ/Δ^; *Trp53* ^Δ/Δ^ PCa. Treatment with anti‐IL‐1β antibody in combination with anti‐PD‐1 antibody further decreased the number of MDSCs (Figure 6E), and sharply upregulated the infiltration of CD8^+^ T cells than anti‐IL‐1β or anti‐PD‐1 antibody treatment alone (Figure 6F). We did not observe significant differences in CD4^+^ T cell numbers under antibody treatment (Figure 6F). Moreover, the RNA levels of *Gzmb* and *Ifng* of CD8^+^ T cells increased significantly under the combination treatment of anti‐IL‐1β antibody and anti‐PD‐1 antibody, indicating a boost of cytotoxic T cell function (Figure 6G).

We also examined the effect of ADT in combination with anti‐IL‐1β antibody and anti‐PD‐1 antibody in another *Pbsn‐Cre4*; *Pten* ^fl/fl^; *Hi‐Myc* organoid‐derived tumor model (Figure S7A, Supporting Information). Although using anti‐IL‐1β antibody alone could not ameliorate tumor growth, the addition of anti‐IL‐1β antibody significantly enhanced the anti‐tumor effect of anti‐PD‐1 antibody (Figure S7B,C, Supporting Information). Consistently, anti‐IL‐1β antibody inhibited the recruitment of MDSCs (Figure S7D, Supporting Information). Blockade of IL‐1β in addition to anti‐PD‐1 antibody further increased the recruitment of CD8^+^ cells (Figure S7E, Supporting Information) and potentiated the expression of *Gzmb* and *Ifng* in cytotoxic CD8^+^ T cells (Figure S7F, Supporting Information). The number of CD4^+^ T cells was not significantly altered by antibody treatment. Altogether, IL‐1β neutralization significantly attenuates the immunosuppressive microenvironment of PCa by suppressing MDSCs. Combination of ADT, anti‐IL‐1β neutralization and anti‐PD‐1 blockade represents an attractive immune therapy strategy for advanced PCa.

## 3 Discussion

**Figure 7:**
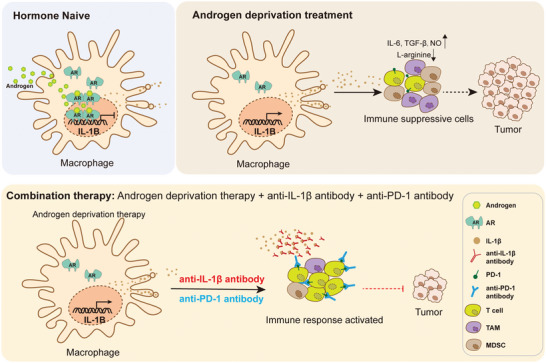
IL‐1β is an androgen‐responsive therapeutic target in PCa‐associated macrophages for immune therapy. Schematic figure describing that IL‐1β is transcriptionally suppressed by AR in PCa‐associated macrophages and upregulated after ADT therapy in PCa. ADT‐mediated secretion of IL‐1β leads to the accumulation of MDSCs and an exacerbation of immune suppressive microenvironment. Targeting IL‐1β and blocking immune checkpoints in combination with ADT can serve as an attractive therapeutic approach for the treatment of advanced prostate cancer.

Our study demonstrates PCa‐associated macrophage as a new androgen‐responsive cell type both in mice and human, in addition to the previously well‐characterized AR‐dependent PCa epithelial cells. We find that AR represses the expression of *IL1B* in TAMs at the transcriptional level. ADT unleashes the binding of AR to the *IL1B* promoter in TAMs, thereby resulting in an enhanced expression and secretion of IL‐1β. Excessive IL‐1β induces massive aggregation of MDSCs, and inhibits the activation of cytotoxic T cells, which causes a further deterioration of the anti‐tumor immune microenvironment. Importantly, IL‐1β blocking antibody coupled with ADT and anti‐PD‐1 antibody exerts a strong anticancer effect on PCa after castration. These results collectively support that immunosuppression by the ADT‐induced aggregation of MDSCs drives PCa progression and points to IL‐1β as an important immune therapy target for advanced PCa in combination with ADT (**Figure** **7**).

ADT is one of the mainstream treatment for PCa.^[^ ^2^ ^]^ Potent antiandrogens such as enzalutamide and abiraterone inhibit the AR pathway in tumor cells,^[^ ^2^, ^3^ ^]^ but the concomitant impact of altered production of cytokines in tumor cells by ADT and effects of AR pathway inhibition in tumor microenvironmental cells also intertwine to lead to a modification of the immune microenvironment.^[^ ^10^, ^25^ ^]^ For example, it has been reported recently that castration results in an AR‐mediated upregulation of IL‐8 in tumor cells to induce the PMN‐MDSC infiltration in Pca.^[^ ^25a^ ^]^ Moreover, ADT is also found to trigger an increased secretion of IL‐23 in MDSCs which promotes therapeutic resistance to androgen deprivation.^[^ ^10b^ ^]^ However, certain discrepancies exist in the findings of impacts of ADT on T cells. AR antagonist enzalutamide and flutamide have been reported to inhibit initial T cell activation and suppress immune responses by repressing the IFN‐*γ* production in T cells.^[^ ^25b^ ^]^ But a separate study reports that AR directly binds to IFN‐*γ* gene to inhibit its transcription, whereas ADT increases IFN‐*γ* production in CD8^+^ T cells and facilitates their cytotoxic effects.^[^ ^26^ ^]^ Recently, studies have also reported that AR regulates the stemness and exhaustion of CD8^+^ T cells through the transcriptional repression of *TCF1/TCF7*.^[^ ^27^ ^]^ In addition, Kissick HT et al. show that ADT induces T‐helper 1 differentiation and promotes T cell infiltration.^[^ ^28^ ^]^ Nevertheless, the prostate cancer is a classic “cold tumor” with limited cytotoxic T cell infiltration.^[^ ^8b^ ^]^ The most abundant immune cell types in PCa from both our own and other studies^[^ ^10^ ^]^ are TAMs and MDSCs. Therefore, we focus our study on AR signaling in TAMs. Intriguingly, the present data analysis from human PCa single cell RNA‐seq shows that AR is most evidently activated in TAMs than other types of immune cells in the PCa. We find that IL‐1β is the most upregulated cytokines in TAMs upon ADT treatment. Promoter‐driven luciferase experiments and ChIP‐q‐PCR experiments support the notion that AR acts as a transcriptional suppressor for IL‐1β. Our work highlights a systemic impact of ADT on TAMs of PCa and reveals a key cytokine that is directly regulated by the AR pathway in TAMs, which provides new insight into our understanding how ADT affects immune cells in the tumor microenvironment.

It is well known that IL‐1β is an important inflammatory mediator.^[^ ^14a^ ^]^ Recently, the role of IL‐1β in solid tumors has gradually been uncovered.^[^ ^16^, ^29^ ^]^ IL‐1β was reported to be upregulated in multiple types of solid tumors,^[^ ^30^ ^]^ and found to stimulate WNT signaling and growth of colon cancer cells,^[^ ^29g^ ^]^ to promote NSCLC by repressing miR‐101 expression through the COX2 / HIF1*α* pathway,^[^ ^29a^ ^]^ to enhance gastric cancer invasion through NF‐*κ*B activation^[^ ^16^, ^29^ ^]^ and to induce the expression of MMP8 in ovarian cancer cells.^[^ ^29f^ ^]^ These works mainly focus on the effect of IL‐1β on tumor cells. In our study, we investigate and present strong experimental evidence for the impacts of IL‐1β on PCa microenvironment. Overexpression of IL‐1β in vivo promotes the recruitment of MDSCs, which can be abrogated by anti‐IL‐1β blocking antibody. Through RNA‐seq analysis and in vitro experiments of treating MDSCs with IL‐1β recombinant protein, we find that IL‐1β induces the aggregation of MDSCs and promotes the expression of MDSC immunosuppressive signals, such as IL6, ARG1 and iNOS. These mechanisms may partially explain the low or no response to immunotherapy in advanced PCa.

As a powerful transcription factor, AR has not only been shown to play a central role in prostate cancer,^[^ ^2^ ^]^ but also has been demonstrated to be a plausible therapeutic target for specific breast cancer subtype^[^ ^31^ ^]^ and hepatocellular carcinoma (HCC). Despite the tumor suppressive effect of AR in ER^+^ breast cancer,^[^ ^32^ ^]^ bicalutamide^[^ ^33^ ^]^ and enzalutamide^[^ ^34^ ^]^ have shown potent clinical anti‐cancer activities in AR^+^ triple‐negative breast cancer (TNBC). Multiple studies demonstrate that AR drives the occurrence of HCC and is associated with the progression and prognosis of HCC.^[^ ^35^ ^]^ However, early clinical trials of antiandrogen therapy for HCC have been disappointing without a clear clinical benefit.^[^ ^36^ ^]^ TAMs are also abundantly recruited to HCC and acts as a key component in promoting HCC pathogenesis.^[^ ^37^ ^]^ It will be interesting to see whether our findings of the adverse effect of ADT on PCa associated macrophages and increase in IL‐1β upon ADT also contribute to the failure of anti‐androgen therapy in HCC. In that case, the combination usage of ADT and anti‐IL‐1β antibody would be a plausible therapeutic approach in a broader range of ADT responsive cancers.

ICB therapies which aim to boost T cell activity display a very low response rate in prostate cancer dominated by immunosuppressive myeloid cells with very few T cells infiltration.^[^ ^8^, ^10^ ^]^ Strategy to target immune modulatory molecules and immunosuppressive cells stands for a conceivable direction to improve ICB efficacy. A combination treatment of anti‐CTLA‐4 antibodies and anti‐PD‐1 antibodies with small molecule cabozantinib or BEZ235 to clear MDSCs has exhibited a good therapeutic effect on PCa in animal models.^[^ ^38^ ^]^ However, cabozantinib and BEZ235 are multi‐kinase inhibitors that not only target MDSCs but also have broad effects on various cells.^[^ ^39^ ^]^ Our work shows that anti‐IL‐1β antibody exert a significant suppression of MDSCs infiltration. In our study, targeting IL‐1β in TAMs synergistically with anti‐PD‐1 antibodies improved immune infiltration in malignant PCa with favorable therapeutic effects on the basis of conventional ADT. Clinically, the anti‐IL‐1β antibody Canakinumab has been approved by the FDA for the treatment of cryopyridine‐associated periodic syndrome (CAPS), tumor necrosis factor receptor‐associated periodic syndrome (TRAPS), hyperimmunoglobulin D syndrome (HIDS) and familial mediterranean fever (MFM) with good safety profiles. Therefore, the clinical translation of anti‐IL‐1β antibody in PCa is practicable. Collectively, our study highlights IL‐1β as an important immune therapy target in advanced PCa and proposes that anti‐IL‐1β antibody together with ADT and ICB is a new attractive combination therapy strategy for PCa.

## 4 Experimental Section

### Patient Samples

The collection of patient samples was approved by the ethics committee of Ren Ji Hospital, School of Medicine, Shanghai Jiao Tong University. The diagnosis of Primary PCa was made based on patients' medical records in the Department of Urology and histological examination by certified pathologists in the Department of Pathology at Ren Ji Hospital. Serum samples from 9 Primary PCa patients and 12 PCa patients after ADT were collected for ELISA to determine serum IL‐1β levels. Three tumor tissues from PCa patients were collected for Immunofluorescent staining. All specimens were obtained with the informed consent of the patients.

### Mice

All mouse experiments were performed according to the protocol approved by the laboratory animal welfare ethics committee of Shanghai Jiao Tong University. *Pbsn‐Cre4*, *Pten* ^fl/fl^, and *Trp53* ^fl/fl^ mouse strains were obtained from JAX lab and crossed as previously reported.^[^ ^40^ ^]^ *Pbsn‐Cre4*; *Pten* ^fl/fl^; *Hi‐Myc* mouse strains were crossed by *Pbsn‐Cre4*, *Pten* ^fl/fl^ mice and *Hi‐Myc* mice which were provided by the NCI mouse repository. C57BL/6J mice were purchased from Shanghai Lingchang Biotechnology Co., Ltd. For the PCa organoid xenograft experiment, 300 organoids were orthotopically transplanted into prostates of 8‐week‐old C57BL/6J mice. Mice were sham‐operated or castrated on day 7. DMSO or enzalutamide was administered by oral gavage daily at a dose of 10 mg kg^−1^ starting from day 10. For antibody treatment, IgG, anti‐IL‐1β antibody, anti‐PD‐1 antibody, or combination of anti‐IL‐1β antibody and anti‐PD‐1 antibody, were administered by intraperitoneal injection at a dose of 8 mg kg^−1^ every two days. Mice were sacrificed on day 21, then prostate tumors and other major organs were carefully harvested, photographed, and processed for subsequent experiments. For IL‐1β‐overexpressed xenograft experiments, 5 × 10^5^ control or IL‐1β‐overexpressed *Pten* ^Δ/Δ^; *Trp53* ^Δ/Δ^ PCa cells were orthotopically transplanted into the prostates of 8‐week‐old C57BL/6J mice. Mice were sacrificed on day 18, then prostate tumors and other major organs were carefully harvested, photographed and processed for subsequent experiments. Information for antibodies used in this study was provided in Table S1, Supporting Information.

### Cell Lines

The Raw264.7 and THP‐1 cell lines were provided by ATCC. Raw264.7 was grown in Dulbecco's Modified Eagle's Medium (DMEM) (Gibco, C11995500BT), while THP‐1 was cultured in RPMI 1640 (Gbico, C11875500BT). Both culture media were supplemented with 10% fetal bovine serum (Gibco, 10270‐106) and 100 U mL^−1^ penicillin and streptomycin (Beyotime, ST488).

### Organoids

The generation of tumor organoids derived from *Pbsn‐Cre4*; *Pten* ^fl/fl^; *Trp53* ^fl/fl^ and *Pbsn‐Cre4*; *Pten* ^fl/fl^; *Hi‐Myc* genetically engineered mice has been described previously.^[^ ^40^ ^]^ Briefly, prostate tumors were harvested, minced, and digested in DMEM containing 0.2 mg mL^−1^ collagenase I (Sangon Biotech, A004194), 0.2 mg mL^−1^ collagenase IV (Sangon Biotech, A004186), 0.01 mg mL^−1^ Dnase I (STEMCELL Technologies, 0 7900), 0.1 mg mL^−1^ dispase (Sangon Biotech, A002100‐0050) and 2% fetal bovine serum at 37 °C for 1.5 h. Then, cells were digested in 0.25% trypsin/EDTA (Sigma, T4049) for 5 min at 37 °C. Single cell suspension was obtained through a 40 µm cell strainer. Cells were blocked with an anti‐CD16/32 antibody (eBioscience, 14‐0161‐86) for 30 min at room temperature, followed by addition of biotin‐conjugated anti‐EpCAM antibody (eBioscience, 13‐5791‐80) for 30 min on ice. Anti‐biotin magnetic beads (MACS, 130‐090‐485) were then added and incubated at 4 °C for 30 min with rotation. The cell suspension was transferred to an LS column (MACS, 130‐042‐401) and passed through a MACS separator to obtain EpCAM^+^ tumor cells. EpCAM^+^ tumor cells were seeded in low‐adsorption 96‐well plates (corning, 3474) in William's E Medium (Gibco, A1217601) supplemented with 10 ng/mL epidermal growth factor (PeproTech, 315‐09‐100), 10 µm Y‐27632 (STEMCELL Technologies, 72 302), 1× Glutamax (Gibco, 35 050), 100 nm DHT (Sigma, A‐8380), 5% charcoal‐stripped FBS (Gemini, 100–119) and 5% matrigel (Corning, 354234) to form organoids.

### Generation of Bone Marrow‐Derived Macrophages and Bone Marrow Myeloid‐Derived Suppressor Cells (BM‐MDSCs)

Bone marrow cells were obtained by flushing the femoral bone marrow cavity of C57BL/6 mice with RPMI 1640 (Gbico, C11875500BT) medium. Red blood cells were removed using red blood cell lysis buffer (Beyotime, C3702) and washed once with PBS. For generation of bone marrow‐derived macrophages (BMDMs), the bone marrow cells were then cultured in RPMI 1640 containing 10% FBS, 10 ng mL^−1^ M‐CSF (Sino biological, 51112‐MNAH) for 7 days to obtain bone marrow‐derived macrophages. For generation of bone marrow myeloid‐derived suppressor cells (BM‐MDSCs), the bone marrow cells were then cultured in RPMI 1640 containing 10% FBS, 20 ng mL^−1^ GM‐CSF (Sino biological, 51048‐MNAH) and 20 ng mL^−1^ IL‐6 (Sino biological, 50136‐MNAE) for 5 days to obtain bone marrow myeloid‐derived suppressor cells.

### ChIP‐seq and ChIP‐qPCR

ChIP‐seq data were obtained from the Gene Expression Omnibus (GSE131381), which contains ChIP‐seq data obtained with AR‐specific antibodies on cell lysates of THP‐1 cells under the treatment of DMSO or R1881.^[^ ^22^ ^]^ ChIP‐seq data were visualized using the Integrative Genomics Viewer.

For ChIP‐qPCR, Raw264.7‐AR overexpressed cells were washed with serum‐free medium and then grown in DMEM medium containing 10% charcoal‐stripped FBS for 72 h. Then, cells were treated with 10 nm DHT or vehicle for 24 h. Next, cells were harvested and AR‐ChIP was performed by SimpleChIP Enzymatic Chromatin IP Kit (Magnetic Beads) (CST, 9003). Briefly, cells were cross‐linked using 1% formaldehyde followed by chromatin isolation. Fragment sizes of 200–700 base pairs were obtained by shearing the chromatin with a sonicator. 10 µL of chromatin preparation was taken as 2% input sample DNA. For immunoprecipitation, 5 µL anti‐AR antibody or control normal rabbit IgG antibody were added to 500 µL chromatin preparation and incubated overnight at 4 °C with rotation. 30 µL Protein G Magnetic Beads (Thermofisher, 88803) were then added to each IP reaction and incubated for 2 h at 4 °C with rotation. Immunoprecipitated protein‐DNA complexes were subjected to stringent washes and eluted from Protein G Magnetic Beads, followed by reverse crosslinking and DNA elution and purification. Putative AR binding chromatin regions predicted by the JASPAR database (https://jaspar.genereg.net/) were examined by qPCR in purified DNA samples. The primers used in ChIP‐qPCR are shown in Table S2, Supporting Information. The qPCR signal from each treatment group was normalized to the signal from the corresponding 2% input samples. The relative abundance of AR binding chromatin regions was calculated by normalizing to the IgG control.

### RNA‐seq and Single Cell RNA‐seq Analysis

Total RNA was extracted from primary prostate tumors of sham‐operated and castrated *Pbsn‐Cre4*, *Pten* ^fl/fl^, and *Trp53* ^fl/fl^ mice, and from *Pten* ^Δ/Δ^; *Trp53* ^Δ/Δ^ orthotopic xenograft tumors (control and IL‐1β OE) using Qiagen RNeasy kit according to the manufacturer's instructions (Qiagen). RNA‐seq libraries were generated by the NEBNext UltraTM RNA Library Prep Kit for Illumina (NEB) and index codes were added to each sample. Cutadap tool was used to cut low‐quality bases at the 3′ end of the reads and sequence adapters. The clean reads were then mapped to the genome (mm10) using Hisat2 (v.2.1.0) to generate SAM files. SAM files were converted to BAM files by SAMtools, and gene counts were then quantified using the Stringtie tool (v.1.3.6). Gene differential expression analysis was performed using DESeq2 (v.1.24.0). Pathways with NES value > 1, normalized *p* value < 0.05, and FDR *q* value < 025% were considered significantly enriched using GSEA software 4.2.0 for GSEA analysis. For TIDE analysis, differentially expressed genes of IL‐1β‐overexpressed tumors compared to control group were mapped to the human genome to assess response to immunotherapy^[^ ^24^ ^]^ (http://tide.dfci.harvard.edu/).

RNA‐seq data on patients with PCa were accessed in dbGaP with accession phs001141.v1.p1, phs000915.v2.p2, and phs000178.v11.p8. AR score was calculated using the GSVA method (v.1.44.5) in R (v.4.1.0) based on ten AR‐responsive genes adopted from a previous publication.^[^ ^41^ ^]^ The correlation of AR score and *IL1B* expression was calculated by ggpubr method (v.0.4.0).

The single cell RNA‐seq data on patients with PCa were accessed in the NCBI Gene Expression Omnibus (GEO) database under accession number GSE137829 and analyzed by Seurat (v.4.1.1) and an R toolkit (https://github.com/satijalab/seurat), using the software R (v.4.1.0). CD45^+^ cell populations are isolated from the scRNA‐seq data and used UMAP^[^ ^42^ ^]^ to visualize the clusters of cells. Clusters were associated with cell types based on the scores of differential expressions of well‐established marker genes for immune cell types: T cells (*CD2*, *CD3D*, *CD3E*, and *CD3G*), macrophages (*CD86*, *CD80*, *iNOS*, *CD163*, and *CD206*), MDSC (*CD14*, and *CD15*), DC (*CD11C*, and *HLADR*), B cells (*CD79A*, *CD79B*, *CD19*, and *MS4A1*), mast cells (*CD117*, and *TRYPTASE*).

### RNA Extraction and Quantitative PCR Analysis

Total RNA from tumor tissue, BMDMs, BM‐MDSCs, Raw264.7 or THP‐1 cell line was extracted by the FastPure Cell/Tissue Total RNA Isolation Kit V2 (Vazyme, RC112‐01). Total RNA from FACS‐purified TAMs, MDSCs, CD8^+^ T cells, and epithelial cells was extracted by the Quick‐RNA Microprep Kit (Zymo, R1051). cDNA was synthesized using the HiScript III All‐in‐one RT SuperMix Perfect for qPCR Kit (Vazyme, R333‐01). qPCR was performed using the ChamQ Universal SYBR qPCR Master Mix Kit (Vazyme, Q711‐02). Gene expression value was normalized to *Actb* as a reference. The primers used in RT‐PCR are shown in Table S2, Supporting Information.

### Plasmids

*AR* expressing plasmid pLENTI6.3/AR‐GC‐E2325 was purchased from Addgene (#82128, deposited by Karl‐Henning Kalland). The *Il1b* promoter (chr2:129370331–129374143,) and *IL1B* promoter (chr2:112836230–112837814) was cloned into Pgl4.17 vector. Primers were provided in Table S2, Supporting Information. Single guide RNA was designed using an online platform (www.benchling.com). The annealed DNA oligos were cloned into pLenti‐CRISPRv2 (Addgene plasmid no. 52961, deposited by F. Zhang) for genome editing. For the cloning of secreted *IL1B*, the signal sequence of *IL‐1RA* was fused to the mature h*IL1B* gene to form a secreted *IL1B* sequence,^[^ ^43^ ^]^ which was then cloned into the plenti‐CMV vector. All plasmids were verified by Sanger sequencing.

### Flow Cytometry

Freshly prepared single cell suspensions were incubated with the Fc blocker anti‐CD16/32 antibody for 30 min at room temperature. Staining antibodies were diluted in PBS supplemented with 2% FBS and cells were incubated for 30 min at room temperature. DAPI was added to cell suspensions to gate live cells. Cell sorting and analysis were performed on BD FACS Aria II flow cytometer and BD LSR Fortessa flow cytometer, respectively. The acquired data were analyzed by FlowJo software (BD). Myeloid derived suppressive cells (MDSCs) were gated as CD45^+^CD11b^+^Gr‐1^+^; tumor associated macrophages (TAMs) were gated as CD45^+^CD11b^+^F4/80^+^; M1‐type TAMs were gated as CD45^+^CD11b^+^F4/80^+^CD86^+^; M2‐type TAMs were gated as CD45^+^CD11b^+^F4/80^+^CD163^+^; CD8^+^ T cells were gated as CD45^+^CD3^+^CD8^+^; CD4^+^ T cells were gated as CD45^+^CD3^+^CD4^+^. Details of the antibodies used are provided in Table S1, Supporting Information.

### Immunofluorescent Staining

Tumor tissues were fixed in 4% PFA for 24 h and then dehydrated in 30% sucrose solution for 24 h. The dehydrated tissues were embedded in OCT (SAKURA, 4583) and frozen at −80 °C for 2 h. Frozen tissues were sectioned at a thickness of 7 µm. Then the sections were placed in the citrate antigen retrieval solution for heat‐induced antigen retrieval. Sections were blocked in 10% donkey serum for 1 h at room temperature, then incubated with primary antibodies overnight at 4 °C, followed by the incubation with secondary antibody for 2 h at room temperature. Sections were then mounted using mounting medium containing DAPI (Vector, H‐1200). Image acquisition was performed at on an Olympus microscope. Details of the antibodies used are provided in Table S1, Supporting Information.

### Immunoblotting

Tissue and cell lysates were prepared in RIPA lysis and extraction buffer (ThermoFisher, 89901) containing the protease inhibitor cocktail (MCE, HY‐K0011). Proteins were separated by SDS‐PAGE and transferred onto polyvinylidene difluoride (PVDF) membranes. Membranes were blocked with 5% non‐fat milk for 2 h at room temperature and then incubated with the indicated diluted primary antibody overnight at 4 °C. Membranes were then applied with horseradish peroxidase (HRP)‐conjugated secondary antibodies for 2 h at room temperature. Protein bands were visualized using immobilon western chemiluminescent HRP Substrate (Millipore, P90720). Details of the antibodies used are provided in Table S1, Supporting Information.

### Luciferase Assay

Plated Raw264.7 or Raw264.7‐AR OE cells were co‐transfected with a firefly luciferase plasmid driven by the *Il1b* promoter (chr2: 129370331–129374143) or *IL1B* promoter (chr2:112836230–112837814) and a Renilla luciferase plasmid driven by the T7 promoter. Transfection was performed using Lipofectamine 3000 (Invitrogen, L3000015) according to the manufacturer's instructions. Following treatment of cells with vehicle or 10 nM DHT or 20 µm enzalutamide (MCE, HY‐70002) for 48 h, cells were harvested and luciferase activity was measured by the Dual‐Glo Luciferase Assay System (Promega, E2920) according to the manufacturer's instructions. Firefly luciferase activity was normalized to the renilla luciferase activity.

### Enzyme‐Linked Immunosorbent Assay

The enzyme‐linked immunosorbent assays were performed by enzyme‐linked immunosorbent assay (ELISA) kit (Elabscience, E‐EL‐H0149) according to the manufacturer's instructions. For examining the concentrations of IL‐1β in serum of PCa patients, 100 µL of serum was used for each sample in the ELISA assays to detect IL‐1β concentrations in the serum of 12 PCa patients after ADT and 9 hormone naive PCa patients. For examining the concentrations of IL‐1β in the lysates and culture media of control and IL‐1β‐overexpressed *Pten* ^Δ/Δ^; *Trp53* ^Δ/Δ^ PCa cells, culture media were collected after culturing cells for 3 days, and cells were resuspended in PBS and then freeze‐thawed three times in liquid nitrogen to obtain cell lysates. Centrifuge culture media and cell lysates at 1000 *g* for 20 min to remove cell debris. 200 µL of supernatants were used in the ELISA assays.

### Statistical Analysis

Data are presented as mean ± SEM. Statistical analysis was performed using Two‐tailed Student's *t* test or Pearson Correlation Coefficient. Significance was determined when the *p*‐value < 0.05.

## Conflict of Interest

The authors declare no conflict of interest.

## Author Contributions

H.H.Z. and W.Q.G. conceived the study; D.W. performed the experiments; X.D., B.D., and W.X. provided clinical patient samples; C.C., K.L., N.J., Y.S., Y.H., and X.X. assisted in animal experiments; P.X., Z.J., and H.Z helped in construction of plasmids; K.Z. participated in immunostaining and imaging; X.C, and J.W. supported data analysis; Y.F. provided Raw264.7 cell line; X.Q. provided support for ELISA experiments; P.Z. helped in experiments design. H.H.Z., W.Q.G., and D.W. interpreted the data and wrote the manuscript.

## Supporting information

Supporting Information

## Data Availability

The data that support the findings of this study are available from the corresponding author upon reasonable request. RNA‐seq data in this study have been deposited to the National Genomics Data Center, China National Center for Bioinformation with Bioproject number PRJCA015342.
