# Supplementary material for: IL‐1β Is an Androgen‐Responsive Target in Macrophages for Immunotherapy of Prostate Cancer
Source: Adv Sci (Weinh). 2023 Apr 24;10(17):2206889. doi: 10.1002/advs.202206889 (PMC10265092; doi:10.1002/advs.202206889)
Supplement: Supplementary file 1 — Supporting Information [file ADVS-10-2206889-s001.pdf]

ADVANCED SCIENCE
Open Access

Supporting Information

for *Adv. Sci.*, DOI 10.1002/advs.202206889

IL-1β Is an Androgen-Responsive Target in Macrophages for Immunotherapy of Prostate Cancer

Deng Wang, Chaping Cheng, Xinyu Chen, Jinming Wang, Kaiyuan Liu, Na Jing, Penghui Xu, Xialian Xi, Yujiao Sun, Zhongzhong Ji, Huifang Zhao, Yuman He, Kai Zhang, Xinxing Du, Baijun Dong, Yuxiang Fang, Pengcheng Zhang, Xueming Qian, Wei Xue, Wei-Qiang Gao and Helen He Zhu**

Supporting Information

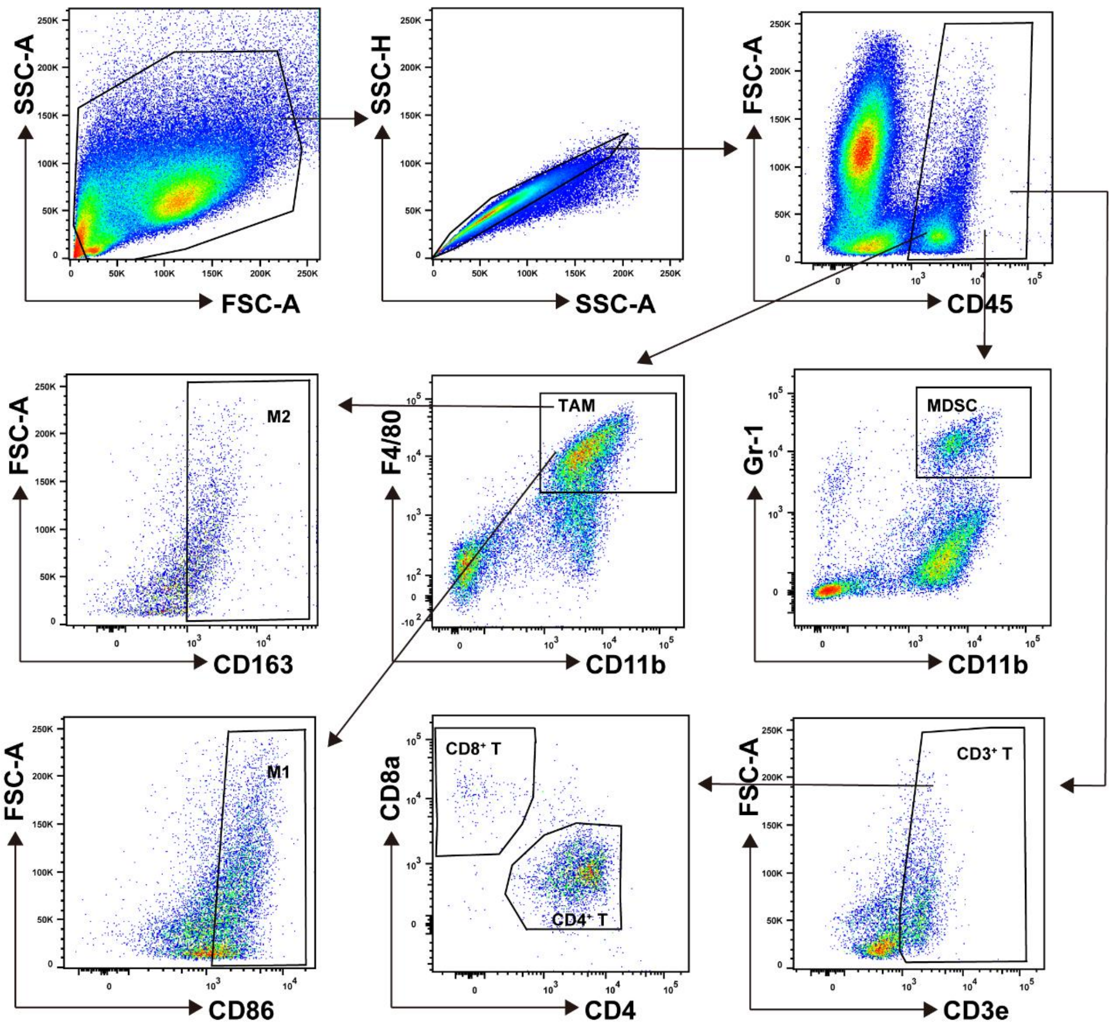

Wang et.al Fig.S1

Figure S1. Gating strategies for analysis of the tumor immune microenvironment by flow cytometry.

Myeloid derived suppressive cells (MDSCs) were gated as $CD45^{+}CD11b^{+}Gr\text{-}1^{+}$; tumor associated macrophages (TAMs) were gated as $CD45^{+}CD11b^{+}F4/80^{+}$; M1-type TAMs were gated as $CD45^{+}CD11b^{+}F4/80^{+}CD86^{+}$; M2-type TAMs were gated as $CD45^{+}CD11b^{+}F4/80^{+}CD163^{+}$; $CD8^{+}$ T cells were gated as $CD45^{+}CD3^{+}CD8^{+}$ and $CD4^{+}$ T cells were gated as $CD45^{+}CD3^{+}CD4^{+}$.

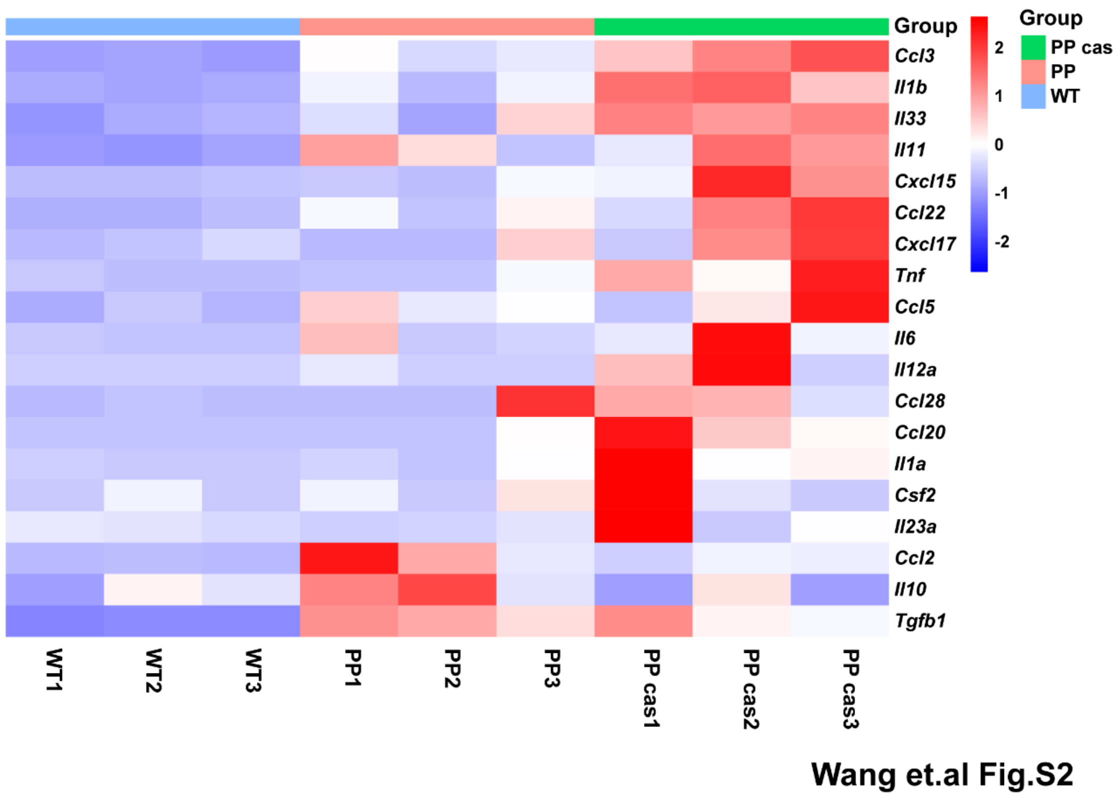

Figure S2. IL-1β is upregulated in PCa after ADT.

Heatmap of differentially expressed genes by RNA-seq of prostate from wild type C57BL/6J mice and prostate tumors from sham or castrated *Pbsn-Cre4; Pten*$^{fl/fl}$*; Trp53*$^{fl/fl}$ mice. WT: wild type mice (n=3); PP: *Pbsn-Cre4; Pten*$^{fl/fl}$*; Trp53*$^{fl/fl}$ mice (n=3); PP cas: castrated *Pbsn-Cre4; Pten*$^{fl/fl}$*; Trp53*$^{fl/fl}$ mice (n=3).

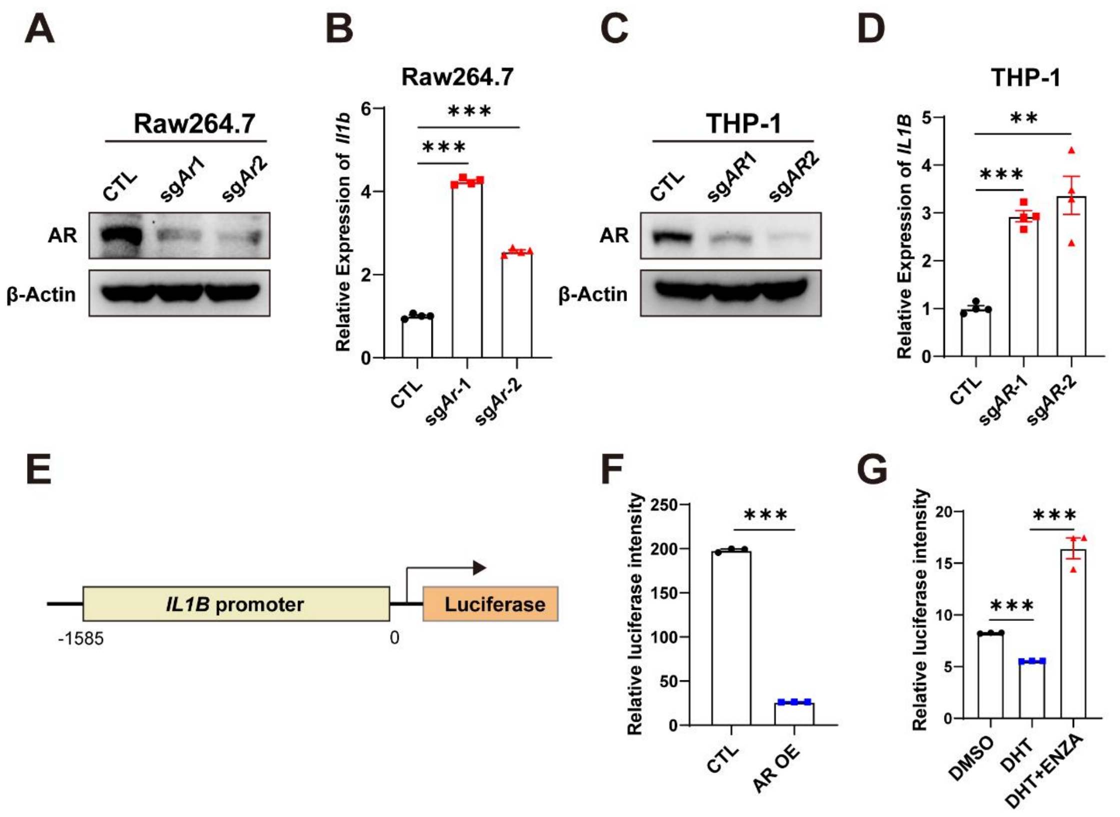

Wang et.al Fig.S3

Figure S3. AR negatively regulates IL-1β in macrophages.

A. Immunoblotting confirmed the efficiency of *Ar* knockout in Raw264.7 cells.

B. qRT–PCR results showing *Il1b* mRNA levels in control and *Ar* knockout Raw264.7 macrophage cell line. Gene expression was normalized to the expression of *Actb*.

C. Immunoblotting confirmed the efficiency of *AR* knockout in THP-1 cells.

D. qRT–PCR results showing *IL1B* mRNA levels in control and *AR* knockout THP-1 macrophage cell line. Gene expression was normalized to the expression of *Actb*.

E. Schematic diagram of IL1B promoter-luciferase reporter constructs.

F, G. Relative luciferase intensity of IL1B promoter-driven firefly luciferase in control and AR OE Raw264.7 cell lines (F), and in Raw264.7 cells treated with

DMSO, 10nM DHT, 10 nM DHT and 10 μM enzalutamide, respectively (G). The firefly luciferase signal was normalized to the co-transfected renilla signal.

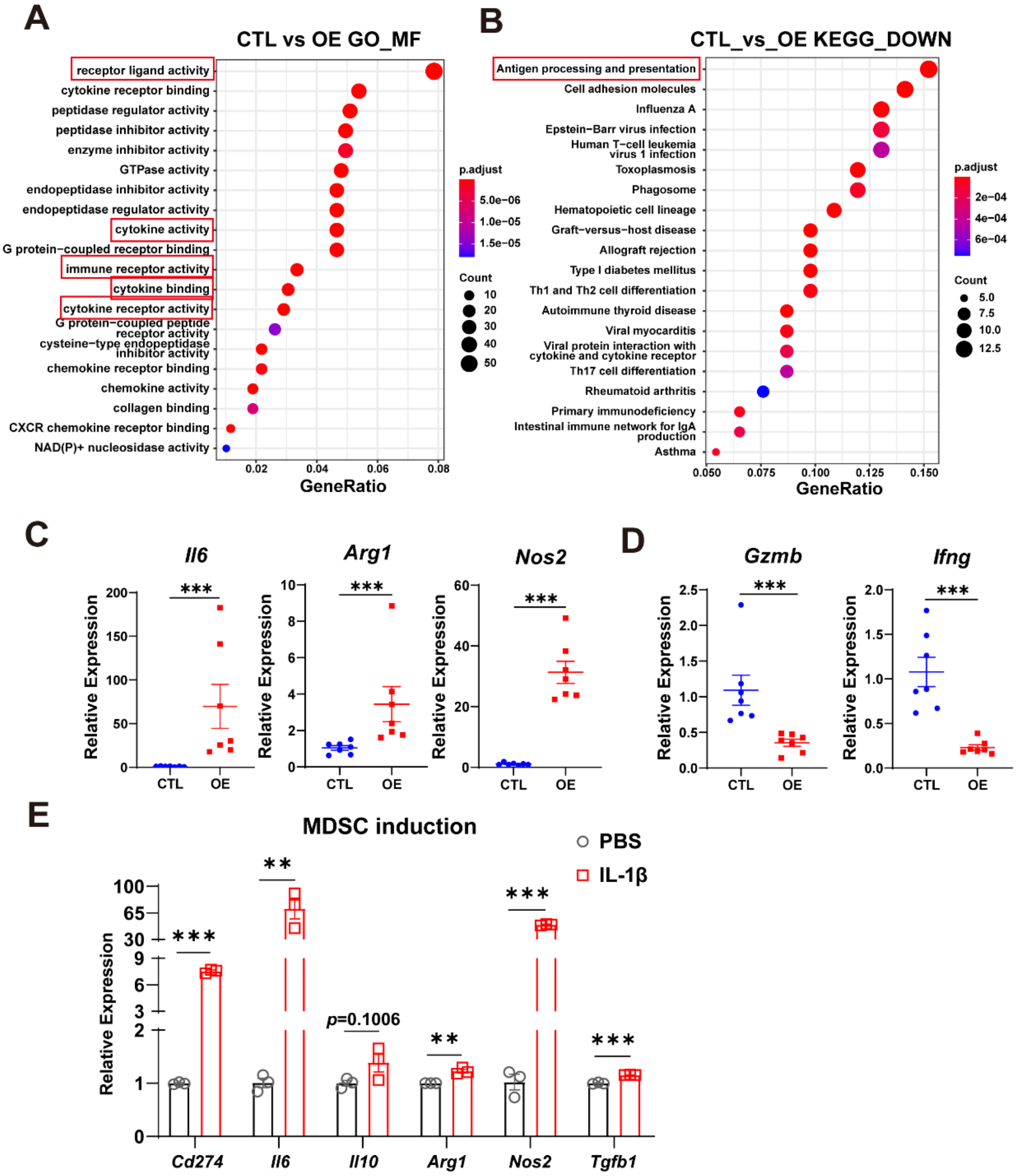

Wang et.al Fig.S4

Figure S4. IL-1β promotes immunosuppressive signaling in MDSCs.

A. Gene sets enriched in IL-1β overexpressed tumors compared to control tumors in GO-molecular function analysis.

B. Gene sets enriched in in IL-1β overexpressed tumors compared to control tumors in KEGG enrichment analysis.

C. qRT–PCR analyses of *Il6*, *Arg1* and *Nos2* mRNA levels in MDSCs sorted from control and IL-1β-overexpressed $Pten^{\Delta/\Delta}$; $Trp53^{\Delta/\Delta}$ prostate tumors. Gene expression was normalized to the expression of *Actb*.

D. qRT–PCR analyses of *Gzmb* and *Ifng* mRNA levels in $CD8^+$ T cells sorted from control and IL-1β-overexpressed $Pten^{\Delta/\Delta}$; $Trp53^{\Delta/\Delta}$ prostate tumors. Gene expression was normalized to the expression of *Actb*.

E. qRT–PCR analyses of *Cd274, Il6, Il10, Arg1, Nos2 and Tgfβ1* in bone marrow derived MDSCs (BM-MDSCs) treated with PBS, 10nM IL-1β for 5 days, respectively.

(Two-tailed Student' s t test was used for the statistical analysis. *, P<0.05; **, P<0.01; ***, P<0.001. Data are presented as means ± SEM.)

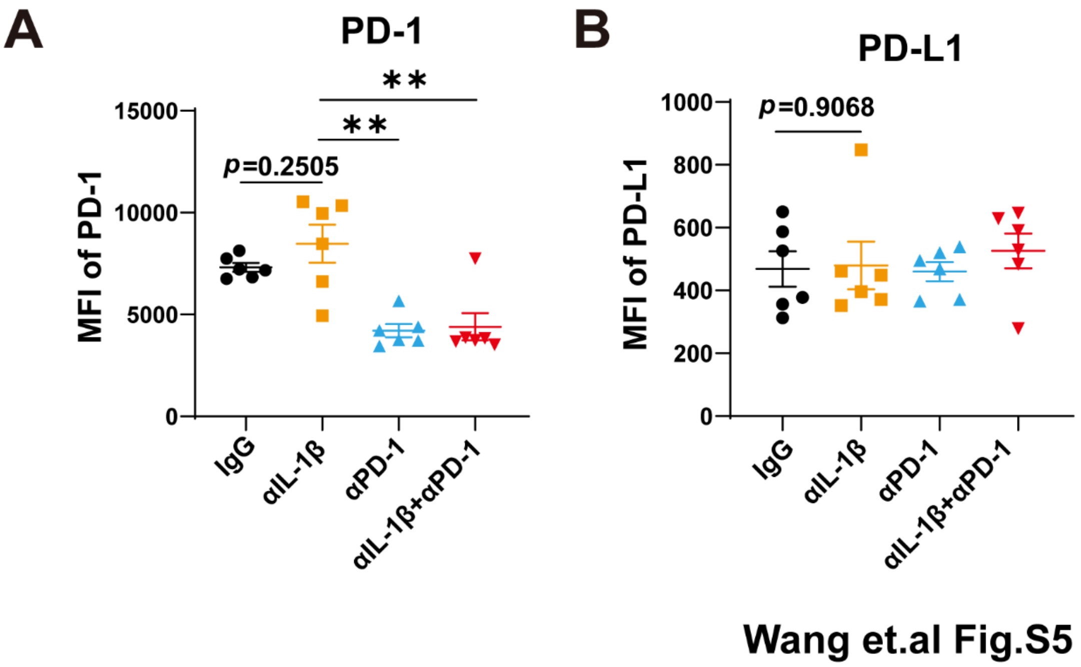

Figure S5. Persistent high expression of PD-1 and PD-L1 in anti-IL-1β antibody-treated PCa tissues.

A, B. Median fluorescence intensity of PD-1 (A) in $CD8^+$ T cells and PD-L1 (B) in prostate tumors of IgG, anti-IL-1β antibody, anti-PD-1 antibody and combinatory antibodies-treated mice.

(Two-tailed Student’ s t test was used for the statistical analysis. *, P<0.05; **, P<0.01. Data are presented as means ± SEM.)

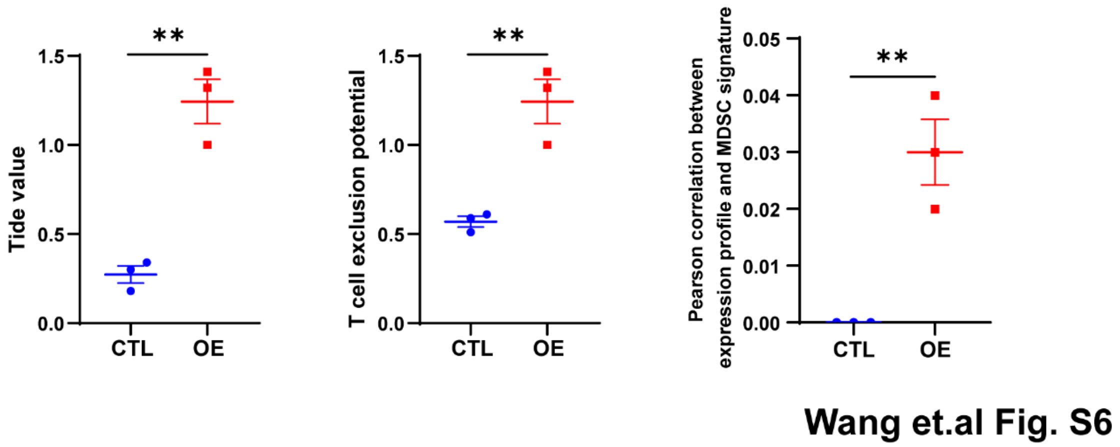

Figure S6. The high expression of IL-1β in prostate cancer is associated with a low response to ICB predicted by TIDE.

TIDE value, T cell exclusion potential and Pearson correlation between expression profile and MDSC signatures analyzed by TIDE tool in control and IL-1β overexpressed PCa tumors.

(Two-tailed Student' s t test was used for the statistical analysis. *, P<0.05; **, P<0.01. Data are presented as means ± SEM.)

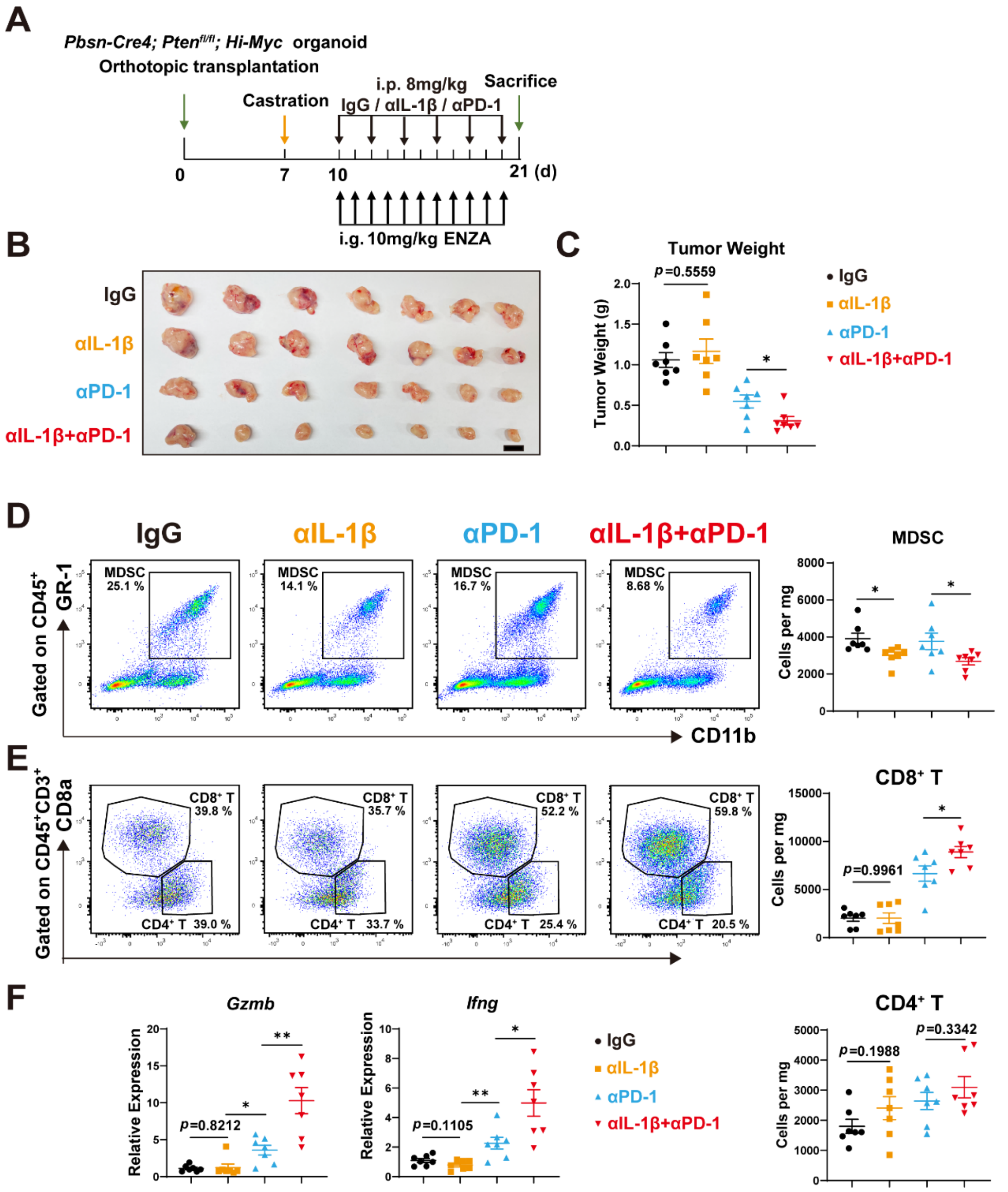

Wang et.al Fig.S7

Figure S7. Androgen deprivation therapy combined with anti-IL-1β and anti-PD-1 immunotherapy inhibits prostate cancer progression in *Pbsn-Cre4; Pten$^{fl/fl}$; Hi-Myc* organoid derived tumors.

A. Schematic illustration of the treatment strategy on *Pbsn-Cre4; Pten$^{fl/fl}$; Hi-Myc* organoid-derived tumors. C57BL/6J mice were castrated on day 7 after implantation of tumor organoids, then treated with enzalutamide (i.g., 10mg/kg) daily from day 10. Mice were arbitrarily divided into four groups and treated with control IgG, anti-IL-1β antibody (i.p. 8mg/kg, every other day), anti-PD-1 antibody (i.p. 8mg/kg, every other day), or anti-IL-1β antibody in combination with anti-PD-1 antibody. Animals were sacrificed on day 21 for analysis and data collection.

B, C. Prostate tumor image (B) (scale bar = 1 cm) and weight (C) of IgG, anti-IL-1β antibody, anti-PD-1 antibody and combinatory antibodies-treated mice.

D, E. Gating strategies for analysis of the tumor infiltrated immune cells by flow cytometry (left panel). Number of MDSCs, $CD8^+$ T and $CD4^+$ T cells normalized to mg of tumor weight (cells per mg) in prostate tumors of IgG, anti-IL-1β antibody, anti-PD-1 antibody and combinatory antibodies-treated mice (right panel).

F. qRT–PCR analyses of *Gzmb* and *Ifng* genes in sorted $CD8^+$ T cells from prostate tumors of IgG, anti-IL-1β antibody, anti-PD-1 antibody and combinatory antibodies-treated mice. Gene expression was normalized to the expression of *Actb*.

(Two-tailed Student' s t test was used for the statistical analysis. *, $P<0.05$; **, $P<0.01$; ***, $P<0.001$. Data are presented as means ± SEM.)

Supplementary Table 1. Antibodies information

Antibodies	Brand	Catalog No.	Dilution and dosage
Recombinant Anti-IL-1 beta antibody	Abcam	ab254360	1:1000 for WB
Recombinant Anti-Androgen Receptor Rabbit monoclonal	Abcam	ab133273	1:200 for IF; 1:1000 for WB
Recombinant Anti-CD68 antibody	Abcam	ab955	1:50 for IF
ACTIN Monoclonal Antibody	ABclonal	AC026	1:3000 for WB
HRP Goat Anti-Rabbit IgG (H+L)	ABclonal	AS014	1:5000 for WB
PerCP anti-mouse CD45.2 Antibody	Biolegend	109826	1:100 for Flow
APC anti-mouse F4/80	Biolegend	123116	1:100 for Flow
FITC anti-mouse/human CD11b	Biolegend	101206	1:100 for Flow
CD19-APC anti-mouse	Biolegend	115512	1:100 for Flow
CD4-Alexa Fluor® 700 anti-mouse	Biolegend	100430	1:100 for Flow
Alexa Fluor® 700 anti-mouse CD86	Biolegend	105024	1:100 for Flow
PE anti-mouse/human CD11b	Biolegend	101208	1:100 for Flow
PE/Cyanine7 anti-mouse CD206	Biolegend	141720	1:100 for Flow
PE anti-mouse CD8a Antibody	Biolegend	100708	1:100 for Flow
FITC anti-mouse CD3ε Antibody	Biolegend	100306	1:100 for Flow
PE anti-mouse Ly-6G/Ly-6C (Gr-1)	Biolegend	108408	1:100 for Flow
CD11c-Alexa Fluor® 700 anti-mouse	Biolegend	117320	1:100 for Flow
Brilliant Violet 421™ anti-mouse CD279 (PD-1)	Biolegend	135221	1:100 for Flow
PE anti-mouse CD163 Antibody	Biolegend	156704	1:100 for Flow
InVivoMab anti-mouse/rat IL-1β	Bioxcell	BE0246	8mg/kg
InVivoMAb anti-mouse PD-1	Bioxcell	BE0146	8mg/kg

(CD279)			
Androgen Receptor (D6F11) XP Rabbit mAb	CST	5153	1:100 for ChIP
F4/80 Monoclonal Antibody (BM8), PerCP-Cyanine5.5	Invitrogen	45-4801-82	1:100 for IF
Donkey anti-Rabbit IgG (H+L) Highly Cross-Adsorbed Secondary Antibody, Alexa Fluor™ 488	Invitrogen	A-21206	1:500 for IF
Donkey anti-Mouse IgG (H+L) Highly Cross-Adsorbed Secondary Antibody, Alexa Fluor™ 594	Invitrogen	A-21203	1:500 for IF
Donkey anti-Rat IgG (H+L) Highly Cross-Adsorbed Secondary Antibody, Alexa Fluor™ 594	Invitrogen	A-21209	1:500 for IF

Supplementary Table 2. Primer and sgRNA information

Primers used in RT-PCR

Primer ID	Primer-F (5' to 3')	Primer-R (3' to 5')
Actb	GGCTGTATTCCCCTCCATCG	CCAGTTGGTAACAATGCCATGT
Arg1	CATTGGCTTGCGAGACGTAGAC	GCTGAAGGTCTCTTCCATCACC
Ccl2	GCTACAAGAGGATCACCAGCAG	GTCTGGACCCATTCCTTCTTGG
Ccl20	GTGGGTTTCACAAGACAGATGGC	CCAGTTCTGCTTTGGATCAGCG
Ccl22	GTGGAAGACAGTATCTGCTGCC	AGGCTTGCGGCAGGATTTTGAG
Ccl28	GTTCATGCAGCATCCAGAGAGC	TCTGAGGCTCTCATCCACTGCT
Ccl3	ACTGCCTGCTGCTTCTCCTACA	ATGACACCTGGCTGGGAGCAAA
Ccl5	CCTGCTGCTTTGCCTACCTCTC	ACACACTTGGCGGTTCCTTCGA
Cd274	GCTCCAAAGGACTTGTACGTG	TGATCTGAAGGGCAGCATTTC
Gzmb	GCTGCTAAAGCTGAAGAGTAAG	CCAGCCACATAGCACACATC
Ifng	AACTCAAGTGGCATAGATGTGGAA	AATGACGCTTATGTTGTTGCTGAT
Il10	CGGGAAGACAATAACTGCACCC	CGGTTAGCAGTATGTTGTCCAGC
Il1a	ACGGCTGAGTTTCAGTGAGACC	CACTCTGGTAGGTGTAAGGTGC
Il1b	TGGACCTTCCAGGATGAGGACA	GTTCATCTCGGAGCCTGTAGTG
Il33	CTACTGCATGAGACTCCGTTCTG	AGAATCCCGTGGATAGGCAGAG
Il6	TACCACTTCACAAGTCGGAGGC	CTGCAAGTGCATCATCGTTGTTC
Nos2	GAGACAGGGAAGTCTGAAGCAC	CCAGCAGTAGTTGCTCCTCTTC
Tgfb1	TGATACGCCTGAGTGGCTGTCT	CACAAGAGCAGTGAGCGCTGAA
Tnfa	CAGGCGGTGCCTATGTCTC	CGATCACCCCGAAGTTCAGTAG

Primers used for ChIP-qPCR

Primer ID	Primer-F (5' to 3')	Primer-R (3' to 5')
BS3	CACAGTAGTCATGGTTATCACAGCA	CATTCCTCCAAGATGGAGGCA

BS4	CTGCCTCCATCTTGGAGGAATG	GGGAACTTTTCAGCTCTTGACTCAC

Primers used for gene cloning

**Primer ID**	**Primer-F (5' to 3')**	**Primer-R (3' to 5')**
Il1b promoter	CGGGGTACCCACAACAGGCTGATTTCTC	GATATCCTCGAGGCTAGCAGCTGCTTCAGACACC
IL1B promoter	GAATTcATCGTGCCACTGCACTCCA	GGCTGCTTCAGACACCTGTGTA

sgRNA sequences

**ID**	**Target sequence (5' to 3')**
sg*AR*-1	AGCAGCAAGAGACTAGCCCC
	CGGCTTAAGCAGCTGCTCCG
sg*AR*-2	CCTCGGTAGGTCTTGGACGG
	TCTCCCCAAGCCCATCGTAG
sg*Ar*-1	GACTTGGGTAGTCTACATGG
	ACCAGGATACCACACTTCGG
sg*Ar*-2	CCCCCATCCAAGACCTATCG
	GGCAGGAGACTAGCCCCCGG
